# Preexisting heart failure with reduced ejection fraction attenuates renal fibrosis after ischemia reperfusion via sympathetic activation

**DOI:** 10.1038/s41598-021-94617-3

**Published:** 2021-07-23

**Authors:** Ryo Matsuura, Tetsushi Yamashita, Naoki Hayase, Yoshifumi Hamasaki, Eisei Noiri, Genri Numata, Eiki Takimoto, Masaomi Nangaku, Kent Doi

**Affiliations:** 1 Department of Nephrology and Endocrinology, Graduate School of Medicine, The University of Tokyo, Tokyo, Japan; 2 Department of Acute Medicine, Graduate School of Medicine, The University of Tokyo, 7-3-1 Hongo, Bunkyo, Tokyo, 113-8655 Japan; 3 Department of Cardiovascular Medicine, Graduate School of Medicine, The University of Tokyo, Tokyo, Japan

**Keywords:** Kidney, Renal fibrosis, Heart failure

## Abstract

Although chronic heart failure is clinically associated with acute kidney injury (AKI), the precise mechanism that connects kidney and heart remains unknown. Here, we elucidate the effect of pre-existing heart failure with reduced ejection fraction (HFrEF) on kidney via sympathetic activity, using the combining models of transverse aortic constriction (TAC) and unilateral renal ischemia reperfusion (IR). The evaluation of acute (24 h) and chronic (2 weeks) phases of renal injury following IR 8 weeks after TAC in C57BL/6 mice revealed that the development of renal fibrosis in chronic phase was significantly attenuated in TAC mice, but not in non-TAC mice, whereas no impact of pre-existing heart failure was observed in acute phase of renal IR. Expression of transforming growth factor-β, monocyte chemoattractant protein-1, and macrophage infiltration were significantly reduced in TAC mice. Lastly, to investigate the effect of sympathetic nerve activity, we performed renal sympathetic denervation two days prior to renal IR, which abrogated attenuation of renal fibrosis in TAC mice. Collectively, we demonstrate the protective effect of pre-existing HFrEF on long-term renal ischemic injury. Renal sympathetic nerve may contribute to this protection; however, further studies are needed to fully clarify the comprehensive mechanisms associated with attenuated renal fibrosis and pre-existing HFrEF.

## Introduction

Clinical observational studies report that chronic abnormalities in cardiac function are a risk factor for acute kidney injury (AKI) development and increasing AKI severity^1,2^. Chronic heart diseases are also inversely associated with renal recovery after AKI^3^. These clinical findings suggest that chronic abnormality in cardiac function induces susceptibility to acute injury and a maladaptive repair process in the kidney. The concept of cardiorenal syndrome suggests that heart and kidney communicate mutually with each other, and the dysfunction in one organ affects the health of the other^4^. This organ interaction was previously recognized as a potential cause of worsening renal function and its poor prognosis has been reported so far^5–7^. Although some possible explanations for the cardiorenal interaction have been proposed, such as venous congestion, activation of the renin-angiotensin aldosterone system, disruption of hypothalamic-pituitary axis, and sympathetic nervous system hyperactivity^8–10^, the precise mechanism still remains unknown partly due to the lack of appropriate animal models that mimic the human clinical features. For exploring potential therapeutic targets against cardiorenal interaction, better animal models are necessary.

Here, to investigate the mechanisms of cardiorenal interaction, we developed a clinically relevant animal model of cardiorenal syndrome by combining transverse aortic constriction (TAC) and renal ischemia reperfusion (IR) for inducing hypertrophic heart failure with reduced ejection fraction (HFrEF) and AKI, respectively. In addition, to evaluate the impact of pre-existing HFrEF on subsequent acute kidney insult, we also examined the impact of pre-existing HFrEF on renal fibrosis progression after IR injury.

## Methods

### Animals and experimental protocol

**Figure 1:**
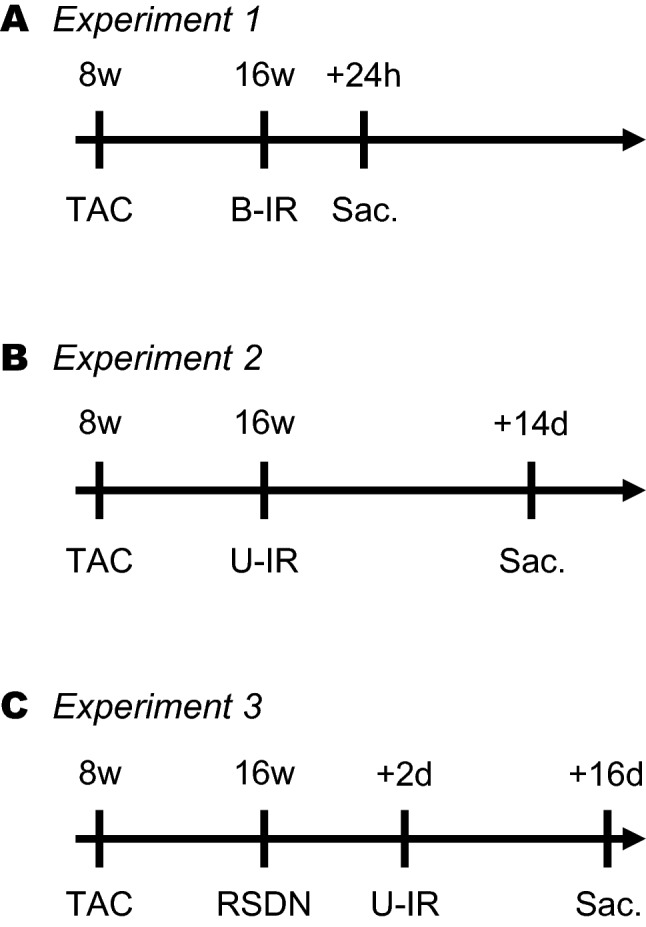
Experimental schematic.

Eight-week-old male C57BL/6 mice were obtained from CLEA Japan (Tokyo, Japan). All experiments were conducted in accordance with the National Institutes of Health’s Guide for the Care, ARRIVE guideline and Use of Laboratory Animals and were approved by The University of Tokyo Institutional Review Board. In Experiment 1, animals were randomly divided into two groups: TAC + bilateral IR group and non-TAC + bilateral IR group. In Experiment 2, animals were also divided into two groups: TAC + unilateral IR group and non-TAC + unilateral IR group. In Experiment 3, animals were divided into four groups: TAC + renal sympathetic nerve denervation (RSDN) + unilateral IR group, TAC + sham + unilateral IR group, non-TAC + RSDN + unilateral IR group, and non-TAC + sham + unilateral IR group. Figure 1 shows the study schematic of the experiments. We conducted all experiments with five to seven animals in each group.

### Surgical procedure

All surgeries were conducted under anesthesia via intraperitoneal injection of ketamine hydrochloride and xylazine hydrochloride. Pressure overload to the heart was produced by constricting the transverse aorta as previously described^11^. The aorta was approached via minimal sternal incision and a 7-0 ligature placed around the vessel using a 27-gauge needle to ensure consistent occlusion. Sham-operated mice underwent the same surgery without constriction. Renal IR with 30-min clamp of bilateral or unilateral (left) renal hilum was performed eight weeks after TAC. Once anesthetized, the mice were placed on a heated surgical table to maintain a temperature of 37 °C throughout surgery. In experiment 3, renal sympathetic nerve denervation (RSDN) was performed two days prior to IR. Briefly, mice were anesthetized, placed in a supine position, the abdominal cavity was exposed, and the bilateral renal arteries and veins were separated from the dorsal wall. The connective tissue surrounding the renal arteries, from the origin of each artery to the renal hilum, which includes the renal sympathetic nerves, were then mechanically delaminated, and peeled away using forceps.

### Echocardiogram

In vivo cardiac morphology was assessed in non-anesthetized mice using transthoracic echocardiography with an ultrasound machine (SONOS 4500; Philips Medical System, Santa Clara, CA). The M-mode left ventricular end-systolic and end-diastolic dimensions, intraventricular septum wall width, and posterior wall width were averaged from 3 to 5 beats. The left ventricular percentage of fractional shortenings was calculated as described in earlier reports^12^.

### Blood chemistry

BUN was measured using the urease indophenol method (Wako Pure Chemical Industries Ltd, Osaka, Japan) on an absorbance 96-well plate reader (Molecular Devices Corp, Sunnyvale, CA) at a wavelength of 570 nm.

### Blood pressure measurement

Blood pressure was measured in a noninvasive tail-cuff method using CODA noninvasive BP system (Kent Scientific Corporation) according to manufacturer’s protocol.

### Measurement of norepinephrine

Kidneys were homogenized in 0.01 N HCl (to charge NoE positively to reduce binding to proteins and optimize solubility) in the presence of EDTA and sodium metabisulfite. After affinity gel extraction, acylation, and enzymatic derivatization, the concentration of norepinephrine were measured using an ELISA kit (LDN, Nordhorn, Germany) according to manufacturer’s protocol as previously described^13^. The NoE ELISA kit includes affinity gel extraction plate, acetylation reagent and enzyme to derivatize NoE.

### Evaluation of norepinephrine turnover

Turnover of NE was calculated on the basis of the disappearance rate of NE after tyrosine hydroxylase inhibition. Animals in each group (sham operated and TAC) were randomly assigned to three subgroups. Mice in the first subgroup were used to establish baseline tissue NE concentrations. These mice were euthanized at time 0. The second subgroup received the tyrosine hydroxylase inhibitor a-methyl-dl-p-tyrosine methyl ester HCl (AMPT, 300 mg/kg ip; Sigma Chemical, St. Louis, MO) at both time 0 and time 4 h and were euthanized at time 8 h. NoE concentration of kidney in both groups were measured as above described. When the log of NoE is plotted against time, the straight line provides the fractional turnover rate.

### Pathological analysis

Collected kidney specimens were fixed in 10% buffered formalin. Paraffin sections of 2 μm thickness were stained with Masson’s trichrome and the interstitial fibrosis area was evaluated in ten randomly selected non-overlapping fields for each section. Slides were visualized at × 400 magnification using ImageJ software (NIH) as previously described^14^.

Immunohistochemical staining of 2 μm paraffin sections was performed using indirect immunohistochemical techniques. After de-paraffinization, antigen retrieval procedures using proteinase K treatment (100 μg/mL) or microwave boiling of tissue for 20 min in 10 mM sodium citrate buffer (pH 6.0) were necessary for all staining experiments. Sections were blocked using 5% goat serum/PBS for 1 h at room temperature, followed by primary polyclonal rat anti-mouse F4/80 macrophage antigen antibody (MCA497R; AbD Serotec, Bio-Rad Laboratories, Kidlington, UK, 1 in 50) or mouse anti- alpha smooth muscle actin (α-SMA) antibody (A2547, Sigma) with overnight incubation at 4 °C for all sections. Subsequently, the endogenous peroxidase activity was blocked with 3% H_2_O_2_/methanol for 20 min. Sections were incubated with biotinylated goat anti-rat polyclonal antibody (Vector Laboratories, 1:100) for 1 h at room temperature and then were incubated with Horseradish Peroxidase Avidin D (Vector Laboratories, 1:200) for 10 min. The peroxidase reaction was carried out by incubating with diaminobenzidine (FUJIFILM Wako Chemicals, Osaka, Japan) and counterstained with hematoxylin. The number of macrophages was counted with DAB and hematoxylin positive cell in X200 fields. The sections were evaluated in double blinded fashion and we confirmed no nonspecific cross-reaction with the secondary antibody. Images were taken on a microscope (Nikon Eclipse 80i, Japan) with a digital camera (Nikon DS-Ri1, Japan).

### Real time PCR

Total RNA was extracted from whole-kidney homogenates using TRIzol (Invitrogen Corp., Carlsbad, CA). Quanti Tect Reverse Transcription Kit (Qiagen Inc,Hilden, Germany) was used to synthesize cDNA from total RNA. Transcripts encoding α-SMA, MCP1, TGF-β, HIF-1, HO-1, CSF2, BMP7 and ALK3 were measured using SYBR Green-based quantitative PCR with the SYBR Green PCR Master Mix (Thermo Fisher Scientific, South San Francisco, CA) and a sequence detection system (ViiA 7; Thermo Fisher Scientific, South San Francisco, CA). In a separate tube, 18S ribosomal RNA (18S rRNA) were amplified using TaqMan quantitative PCR. We evaluated transcript level of each gene with the Standard-Curve method with normalizing the relative expression of the genes of interest with respect to 18S rRNA.

### Statistical analyses

The results of the statistical analyses are expressed as mean ± SDs. Differences between experimental groups were determined using Mann Whitney U test because normal distribution of data were not confirmed. p < 0.05 was considered as statistically significant. All calculations were conducted using JMP 14.0 software (SAS Institute Inc., Cary, NC).

## Results

### Pre-existing HFrEF had no impact on acute renal insult by ischemia reperfusion

**Figure 2:**
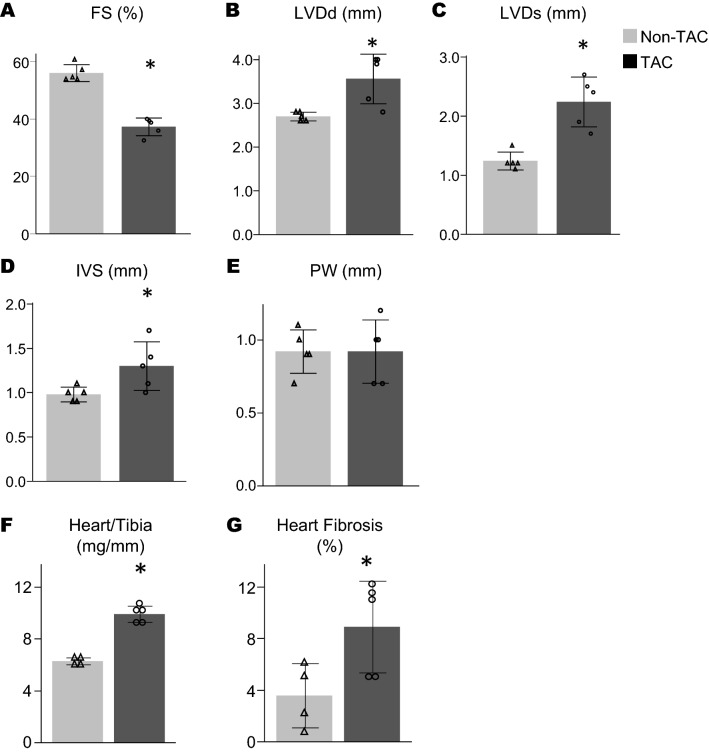
Characteristics of TAC mice. This Figure shows (**A–E**) the echocardiogram result, (**F**) the weight of heart measured as heart/tibia ratio, and (**G**) the area of heart fibrosis. The echocardiogram revealed that TAC mice had reduced fractional shorting (**A**), larger left ventricular end-diastolic and end-systolic diameter (LVDd, LVDs) and thickened interventricular septum (IVS) (**B–D**). Significant differences were observed in heart weight and fibrosis between the TAC mice and the non-TAC mice (**F,G**). *p < 0.05. Gray bar and open triangle, non-TAC mice; black bar and open circle, TAC mice. All graphs were created using JMP 14.0 software (SAS Institute Inc., Cary, NC).

**Figure 3:**
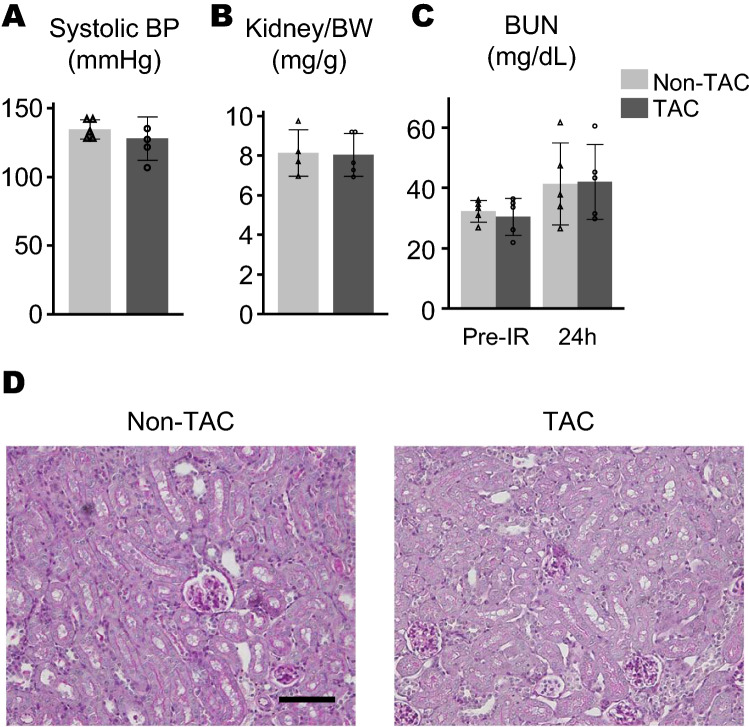
TAC had no effect on the acute phase after renal IR (Experiment 1). (**A**) Systolic blood pressure before renal IR, (**B**) kidney weight per body weight (BW) 24 h after renal IR, and (**C**) BUN before and 24 h after renal IR were similar between the non-TAC (n = 5) and the TAC groups (n = 5). Gray bar, non-TAC mice; black bar, TAC mice. (**D**) Representative image of renal pathology at 24 h after renal IR in PAS staining. (Bar = 100 μm). All graphs were created using JMP 14.0 software (SAS Institute Inc., Cary, NC).

HFrEF was induced by TAC. Ultrasound examination 8 weeks postoperative of TAC procedure revealed a reduction of fractional shorting (FS), dilated end-diastolic and end-systolic diameter of left ventricle, and thickened left ventricular wall (Fig. 2A–E). Increased heart weight and development of fibrosis were confirmed in the TAC mice, but not in the non-TAC mice (Fig. 2F,G). Systemic blood pressure and kidney weights in both groups were found to be similar (Fig. 3A,B). Acute renal insults were evaluated 24 h after 25 min bilateral renal IR in TAC and non-TAC mice (Experiment 1, Fig. 1A). Examination of BUN concentrations and renal pathology found that no significant differences were observed between TAC and non-Tac mice (Fig. 3C,D).

### Renal fibrosis after ischemia reperfusion was reduced in the TAC mice

**Figure 4:**
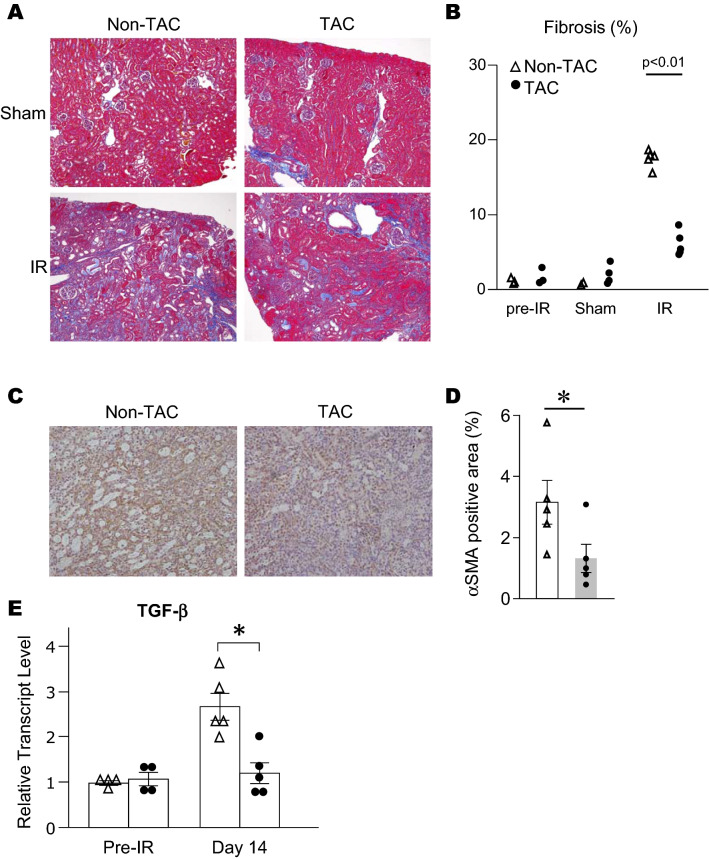
Renal interstitial fibrosis development after IR (Experiment 2). (**A**) Representative images of renal pathology with Masson Trichrome staining 14 days after IR. (**B**) Quantitative analysis of renal fibrotic areas (n = 4–5 per group). (**C**) Representative images of α-SMA staining 14 days after IR. (**D**) Quantitative analysis of α-SMA positive area (n = 4–5 per group). (**E**) mRNA levels of TGF-β is shown (n = 4–5 per group). *p < 0.05. Open triangle, non-TAC mice; filled circle, TAC mice. All graphs were created using JMP 14.0 software (SAS Institute Inc., Cary, NC).

**Figure 5:**
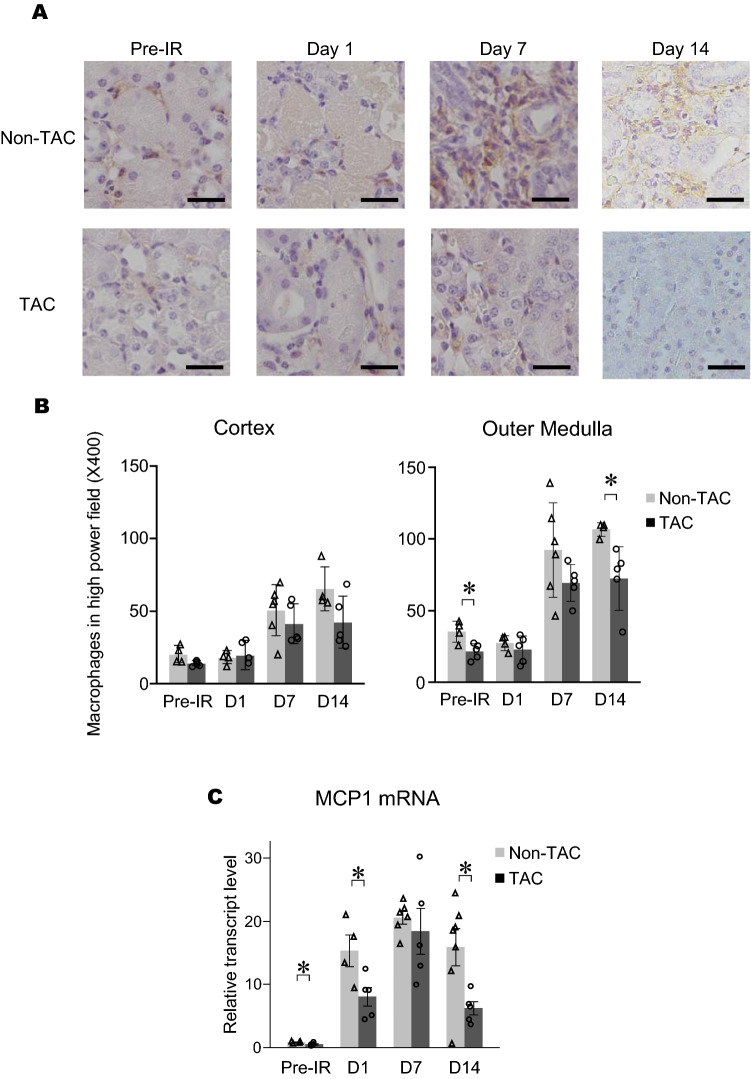
Macrophage infiltration and MCP1 mRNA expression after IR (Experiment 2). (**A**) Representative images of immunohistochemistry for F4/80 before, at 1, 7 and 14 days after IR (Bar = 25 μm). (**B**) Number of F4/80 positive cells in high power field (× 400) and (**C**) MCP1 mRNA expression (n = 4–5 per group). *p < 0.05. Gray bar and open triangle, non-TAC mice; black bar and open circle, TAC mice. All graphs were created using JMP 14.0 software (SAS Institute Inc., Cary, NC).

No significant impact of TAC on acute renal insult prompted us to evaluate whether subsequent renal fibrosis progression would be affected by pre-existing HFrEF. Thus, in Experiment 2, the formation of renal fibrosis was evaluated 2 weeks after 30-min unilateral renal IR in the TAC and the non-TAC mice. Systemic blood pressure and kidney weights in both groups were similar (Supplemental Fig. S1A,B). Histological analyses of the fibrotic areas revealed that interstitial fibrosis of the kidneys in the TAC mice was significantly milder than in the non-TAC mice (TAC, 6.4 ± 1.7% vs non-TAC, 17.4 ± 1.2%, Fig. 4A,B). The levels of expression of α-SMA was also significantly increased in the kidneys of TAC mice (Fig. 4C,D). Transforming growth factor beta (TGF-β) mRNA expression was higher in the TAC mice than the non-TAC mice (Fig. 4E). In addition, macrophage infiltration evaluated by immunohistochemistry for F4/80, a mouse macrophage marker, revealed that macrophage populations in the kidney were increased in non-TAC mice compared to TAC mice (Fig. 5A,B). Monocyte chemoattractant protein 1 (MCP1) mRNA expression in the TAC mice was significantly lower than in the non-TAC mice (Fig. 5C, Supplemental Table S1). It should be noted that the kidney of TAC mice showed mild but significantly lower macrophage infiltration and MCP-1 expression before induction of renal IR.

**Figure 6:**
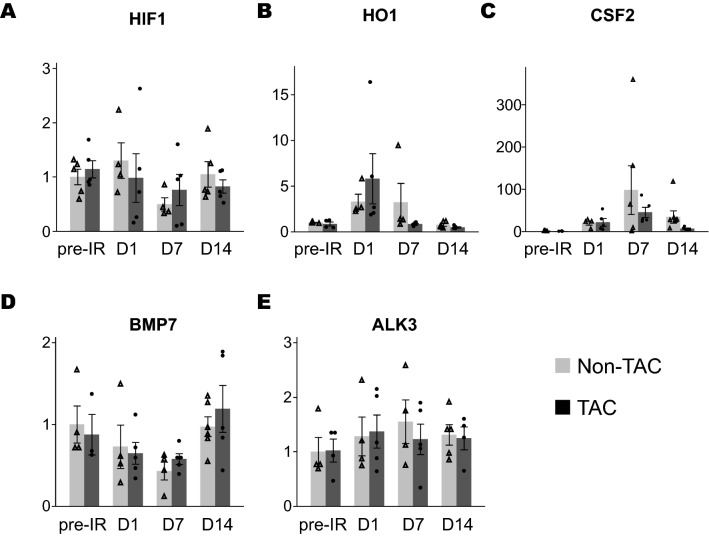
Relative transcript level of genes related with anti-fibrotic pathway (Experiment 2). mRNA levels of (**A**) HIF1, (**B**) HO-1, (**C**) CSF2, (**D**) BMP7 and (**E**) ALK-3 in the kidneys of non-TAC and TAC mice are shown (n = 4–5 per group) after IR. Gray bar and open triangle, non-TAC mice; black bar and filled circle, TAC mice. All graphs were created using JMP 14.0 software (SAS Institute Inc., Cary, NC).

Several previously reported protective genes (hypoxia inducible factor 1, HIF-1α; heme oxygenase 1; HO-1, colony-stimulating factor 2, CSF-2; bone morphogenetic protein-7, BMP-7; and activin-like kinase 3; ALK3) against renal fibrosis progression after ischemia reperfusion were evaluated after IR. However, we found no significant differences in the expression of these genes in the kidneys of TAC and non-TAC mice (Fig. 6).

### Effect of sympathetic activation on renal fibrosis after ischemia reperfusion in TAC mice

**Figure 7:**
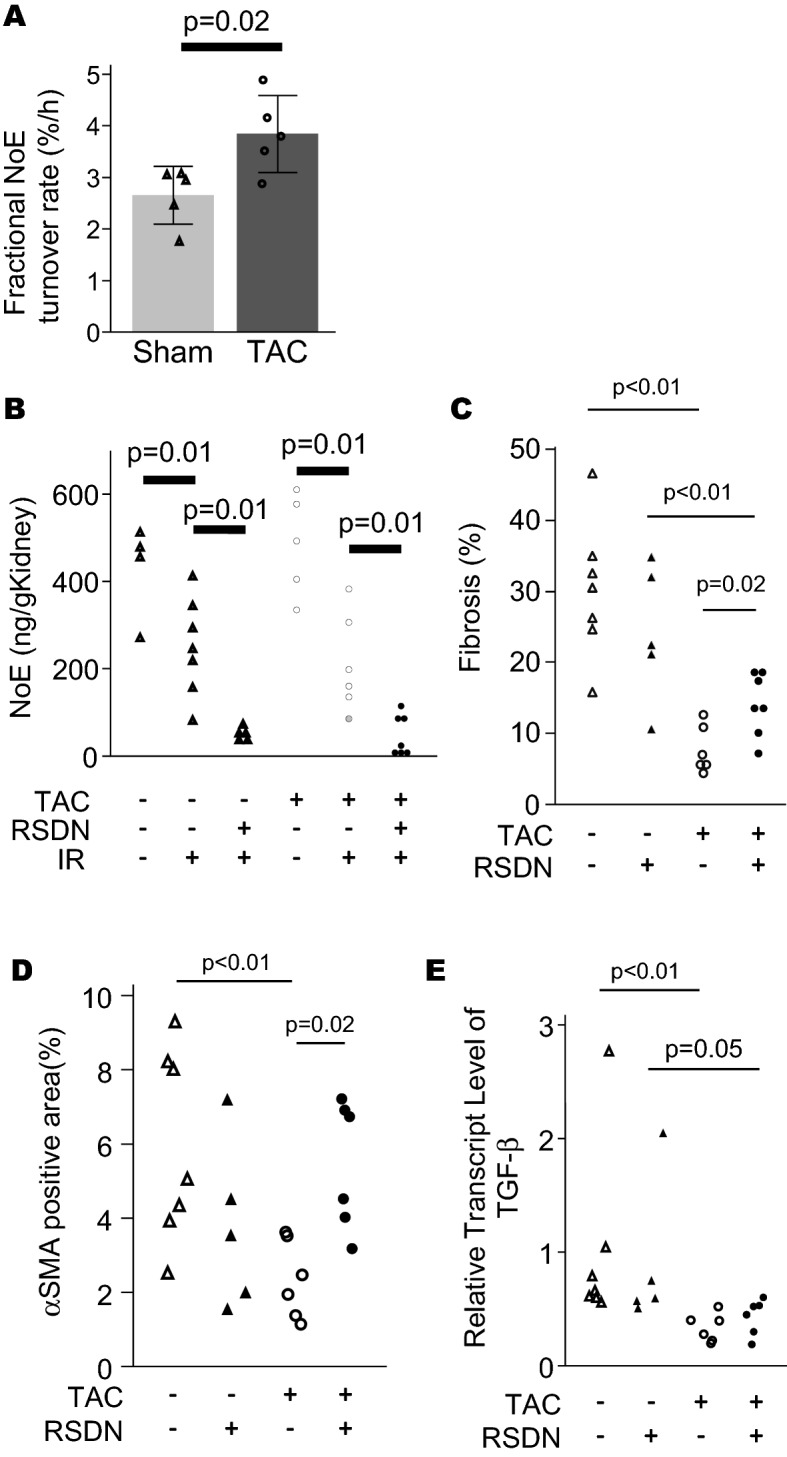
Renal sympathetic denervation worsened renal fibrosis after IR in TAC mice (Experiment 3). (**A**) Fractional Norepinephrine (NoE) turnover rate (%/h), (**B**) NoE concentration in kidney (ng/g kidney), (**C**) Quantified renal fibrotic areas and (**D**) α-SMA positive area, and (**E**) mRNA level of TGF-β are shown (n = 5–7 per group). ^#^p < 0.05. In (**B**), open triangle represents non-TAC mice without RSDN and IR; gray triangle non-TAC + IR mice without RSDN; filled triangle non-TAC + IR with RSDN; open circle, TAC mice without IR and RSDN; gray circle, TAC + IR mice without RSDN; filled circle, TAC + IR + RSDN mice. In (**C–E**), open triangle represents non-TAC + IR mice without RSDN; filled triangle non-TAC + IR with RSDN; open circle, TAC + IR mice without RSDN; filled circle, TAC + IR + RSDN mice. All graphs were created using JMP 14.0 software (SAS Institute Inc., Cary, NC).

We confirmed the sympathetic activation in HFrEF model by the result that TAC mice had a higher NoE turnover rate than non-TAC mice though NoE concentration in kidney were not significantly different (Supplemental Fig. S7A,B). To evaluate whether sympathetic activation affects renal fibrosis progression after IR, renal sympathetic denervation (RSDN) was conducted. Norepinephrine concentration in the kidneys was partially reduced by IR in the TAC and the non-TAC groups [483.6 ± 115.4 ng/g tissue (TAC-sham, n = 5) vs 244.4 ± 178.9 ng/g tissue (TAC-IR, n = 7), p < 0.05, 429.9 ± 108.8 ng/g tissue (non-TAC-sham, n = 4) vs 250.1 ± 112.0 ng/g tissue (non-TAC-IR, n = 7), p < 0.05]. The RSDN procedure remarkably reduced renal tissue norepinephrine (Fig. 7A), while plasma norepinephrine concentrations were similar in all the groups (Supplemental Fig. S2). RSDN did not affect blood pressure, plasma UN and kidney weights (Supplemental Fig. S3).

Histological analyses of fibrotic areas found in the kidneys revealed that renal interstitial fibrosis was attenuated by the TAC procedure regardless of RSDN (non-TAC 30.1 ± 9.5%, non-TAC + RSDN 24.1 ± 9.6%, TAC 7.6 ± 3.4%, TAC + RSDN 14.0 ± 4.3%). On the other hand, RSDN increased renal fibrosis and α-SMA positive area in the TAC group, and importantly, had no impact on renal interstitial fibrosis and α-SMA positive was observed in the non-TAC group with RSDN (Fig. 7B,C). TGF-β mRNA expression in the TAC group was significantly lower than in the non-TAC groups, but we found that RSDN did not affect TGF-β mRNA expression (Fig. 7D).

## Discussion

This study showed the significant impact of pre-existing HFrEF on renal fibrosis progression induced by acute IR insults. The obtained data suggest a protective role of sympathetic nerve activity on this organ crosstalk between the heart and the kidney. Of note, several unexpected observations that TAC had no effect on the acute phase after renal IR and renal fibrosis following IR was significantly reduced in TAC mice were also obtained in this study.

Recent clinical studies found that heart disease is one of risk factors for AKI^1,2^. Activation of sympathetic nerves, the renin–angiotensin–aldosterone system, and venous congestion are all considered to have negative effects on kidney^8^. Indeed, previous basic research revealed that sympathetic denervation attenuated tubular injury and renal fibrosis after unilateral ureteral obstruction or IR^15,16^. Sympathetic denervation also has a protective effect in a model of CKD^17^. A clinical study found that elevated central venous pressure was associated with worsening renal function in heart failure^18^, which suggests that venous congestion can cause renal injury. These basic and clinical results led us to the hypothesis that tubular injury and renal fibrosis would worsen in TAC mice. However, in our study, TAC did not have an effect on acute injury and had a significant protective effect on the subacute phase after IR.

In other studies, TAC induces sympathetic activation^19^ and administration of long-acting β stimulator promotes renal recovery after renal IR^20^. Another study found that sympathetic activation under restraint stress protects against renal IR^21^. Thus, we assumed that sympathetic activity would contribute to abrogated renal fibrosis in TAC mice. This study also revealed that sympathetic denervation deteriorated renal fibrosis after IR, which suggests an association of sympathetic activity with recovery from renal ischemia, consistent with the findings of previous studies^20,21^. However, we should take into consideration our finding that TGF-β mRNA expression was not affected by RSDN, whereas renal fibrosis in the TAC mice was deteriorated by RSDN. Although TGF-β is one of the most common genes associated with fibrosis^22,23^, the reduced fibrosis by sympathetic activation may be involved in other fibrotic pathways. Further investigation will be necessary to clarify the precise mechanisms of renal protection by sympathetic activation in the condition of HFrEF.

Although sympathetic activation is assumed to be a contributor to enhancing renal recovery after IR under pressure overload on the heart, it should be noted that attenuation of renal fibrosis by pre-existing HFrEF by TAC was observed in RSDN mice. These data indicate that mechanisms other than sympathetic activation are involved. For example, it is well known that HIF-1 and HO-1, which are activated in hypoxic state, have a protective effect against IR injury in kidney^24–27^. A recent study also showed that CSF-2, secreted by tubular cells after IR, promotes tubular proliferation via STAT5-dependent macrophage activation and may enhance renal recovery^28^. However, in our experiment, expression of these genes did not show a significant difference in TAC mice versus non-TAC mice. Additionally, although BMP-7 and its receptor ALK3, are known to be key players in the anti-fibrotic pathway after AKI^29^, the result of BMP-7 and ALK3 mRNA expression also indicated that these genes are not related in the pathophysiology of enhanced renal recovery after IR by TAC (Fig. 7).

Mild but significant differences regarding macrophage infiltration and MCP-1 expression before induction of renal IR have been observed between TAC and non-TAC mice and TAC reportedly induced a transient increase of MCP-1 expression in the heart within one week after TAC surgery^30,31^. However, in this study we found not an increase but a decrease of MCP-1 and macrophage infiltration in the kidney 8 weeks after TAC surgery. Decreased macrophage influx to the kidney might be partly explained by the protective effect of pre-existing HFrEF against renal fibrosis development. Mechanisms other than sympathetic activity should be investigated further.

Another factor that should be considered in cardiorenal syndrome is the hemodynamic state. Recent animal studies revealed that the decrease of renal blood flow (RBF) is associated with fibrosis development after exposure of acute renal ischemic insult^32^. Prophylactic administration of angiotensin receptor blocker (ARB) for renal IR did not cause any impact in acute phase, however glomerular filtration rate (GFR) reduction and renal fibrosis development 9 months later were prevented by ARB treatment^33^. RBF and intraglomerular pressure seem to be associated with renal fibrosis after ischemic AKI. A limitation of this study was that it was unclear whether HFrEF induced by TAC had any impact on RBF and GFR, though we found that blood pressure in TAC mice was not significantly different from non-TAC mice.

There are some limitations in this study. First, we adopted different IR models in acute and chronic experiments. Bilateral and unilateral IR may be different in compensation of contralateral kidney in unilateral IR, although previous studies used unilateral IR for AKI to CKD model^34–36^. Second, this study did not conduct the experiment of sympathetic nerve activation, that would demonstrate the hypothesis that sympathetic activation leads to tubular regeneration via macrophage activation and tubular proliferation. Third, there is a possibility that renal injury following IR was so moderate in acute experiment that we could not have detected the influence of HFrEF. Further evaluation with more severe IR is necessary. Fourth, sample size in each experiment was small. Because this study required repeated surgical procedures to the animals, it was difficult to increase the number of animals in each group.

In conclusion, pre-existing HFrEF induced by TAC attenuated renal fibrosis after acute IR injury, while no significant difference was observed in acute phase or renal insult. Sympathetic nerve activation may have a role in suppressing renal fibrosis development. Further studies are needed to investigate the comprehensive mechanisms involved with pre-existing HFrEF attenuated renal fibrosis.

## Supplementary Information

Supplementary Information.
