# Supplementary material for: Preexisting heart failure with reduced ejection fraction attenuates renal fibrosis after ischemia reperfusion via sympathetic activation
Source: Sci Rep. 2021 Jul 23;11:15091. doi: 10.1038/s41598-021-94617-3 (PMC8302613; doi:10.1038/s41598-021-94617-3)
Supplement: Supplementary file 1 — Supplementary Information. [file 41598_2021_94617_MOESM1_ESM.pdf]

Preexisting heart failure with reduced ejection fraction attenuates renal fibrosis after ischemia reperfusion via sympathetic activation

Ryo Matsuura[1], Tetsushi Yamashita[1], Naoki Hayase[2], Yoshifumi Hamasaki[1], Eisei Noiri[1], Genri Numata[3], Eiki Takimoto[3], Masaomi Nangaku[1,] Kent Doi[2]

Table of Contents

Supplemental Figure 1. Plasma UN and kidney weight in Experiment 2...................................2
Supplemental Figure 2. Plasma norepinephrine concentration. 3
Supplemental Figure 3. Kidney weight, plasma UN and blood pressure in Experiment 3..........4
Supplemental Table 1. Transcript level of MCP1 in Experiment 2. 5

Supplemental Figure 1. Plasma UN and kidney weight in Experiment 2.

The graph shows (A) plasma UN and (B) kidney weight two weeks after IR in non-TAC and TAC mice.

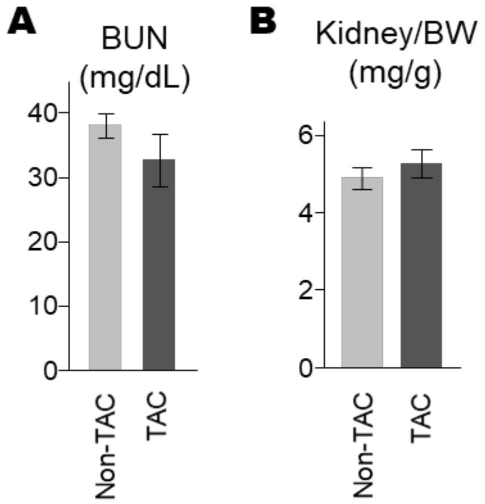

Supplemental Figure 2. Plasma norepinephrine concentration.

Plasma norepinephrine concentration in each group is shown. Plasma norepinephrine concentrations levels in all the groups were not significantly different.

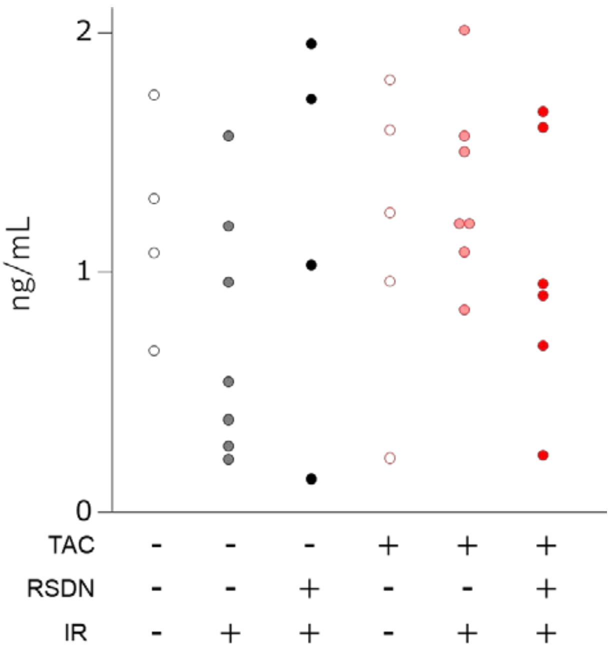

Supplemental Figure 3. Kidney weight, plasma UN and blood pressure in Experiment 3.

The graph shows (A) blood pressure before IR, plasma UN and (B) kidney weight two weeks after IR in non-TAC and TAC mice. These parameters were not significantly different.

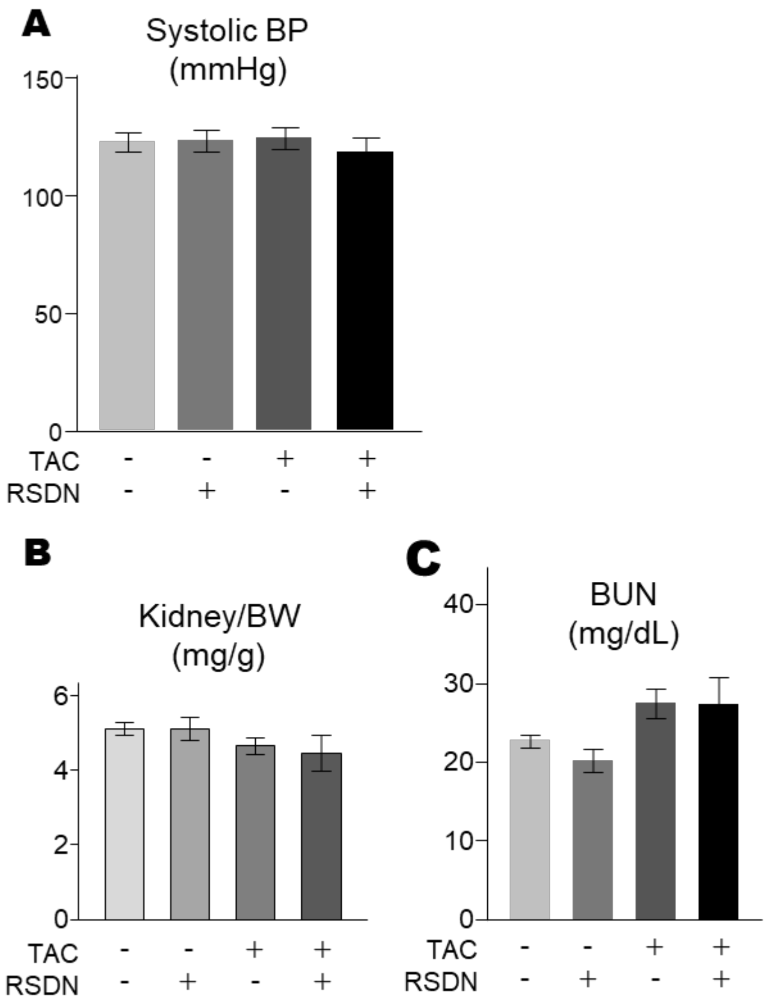

Supplemental Table 1. Transcript level of MCP1 in Experiment 2.

This table is related to Figure 5C. Panel A shows the mean and standard deviation of MCP1 transcript level. Panel B shows all individual data on MCP1.

A. Summary of MCP1 transcript level.

	Pre-IR	Day 1	Day 7	Day 14
non-TAC	1.00±0.33	15.3±5.01	20.5±2.47	15.9±7.77
TAC	0.53±0.24	8.04±3.29	18.4±8.13	6.23±2.36

B. Individual data on MCP1

Group	Day	MCP1 transcript level
Non-TAC	Pre-IR	1.625025581
Non-TAC	Pre-IR	0.975199856
Non-TAC	Pre-IR	1.227148594
Non-TAC	Pre-IR	0.913510148
Non-TAC	Pre-IR	0.868899567
Non-TAC	Pre-IR	0.627253034
Non-TAC	Pre-IR	0.76296322
TAC	Pre-IR	0.871175922
TAC	Pre-IR	0.855286346
TAC	Pre-IR	0.361263701
TAC	Pre-IR	0.266167286
TAC	Pre-IR	0.47654628
TAC	Pre-IR	0.516445707
TAC	Pre-IR	0.394527037
Non-TAC	Day 1	13.38583213
Non-TAC	Day 1	17.50752034
Non-TAC	Day 1	9.402464972
Non-TAC	Day 1	20.964754
TAC	Day 1	12.44944806
TAC	Day 1	9.083155967
TAC	Day 1	9.107223831
TAC	Day 1	4.455440322
TAC	Day 1	5.095449489
Non-TAC	Day 7	19.34332675

Non-TAC	Day 7	22.00058056
Non-TAC	Day 7	23.51671142
Non-TAC	Day 7	21.34990981
Non-TAC	Day 7	20.63043343
Non-TAC	Day 7	16.3774085
TAC	Day 7	30.18066754
TAC	Day 7	22.76909414
TAC	Day 7	12.92118112
TAC	Day 7	9.94092069
TAC	Day 7	16.16465713
Non-TAC	Day 14	18.49006076
Non-TAC	Day 14	0.583954975
Non-TAC	Day 14	19.01442844
Non-TAC	Day 14	12.08557111
Non-TAC	Day 14	20.80427615
Non-TAC	Day 14	15.85215248
Non-TAC	Day 14	24.39590142
TAC	Day 14	9.688012439
TAC	Day 14	3.651981022
TAC	Day 14	6.467464045
TAC	Day 14	4.441619396
TAC	Day 14	6.877231836
